# School Climate and Black Adolescents’ Psychological Functioning: The Roles of Parental Self-Efficacy and Parenting Practices

**DOI:** 10.3390/bs15070933

**Published:** 2025-07-10

**Authors:** Fatima A. Varner, Sophia J. Lamb, Hin Wing Tse, Ahniah R. Charles, Naila A. Smith, Sheretta T. Butler-Barnes

**Affiliations:** 1 Department of Human Development and Family Sciences, The University of Texas at Austin, Austin, TX 78712, USA; slamb@utexas.edu (S.J.L.); florence.tse@utexas.edu (H.W.T.); ahniahc@utexas.edu (A.R.C.); 2 School of Education and Human Development, The University of Virginia, Charlottesville, VA 22903, USA; nailasmith@virginia.edu; 3 Brown School, Washington University in St. Louis, St. Louis, MO 63130, USA; sbarnes22@wustl.edu

**Keywords:** school climate, adolescents, psychological functioning, parenting, parental self-efficacy, Black Americans

## Abstract

Based on ecological systems theory, adolescents’ school climates can influence family interactions. In this study, it was tested whether associations between adolescents’ and parents’ perceptions of school climate in 7th grade (Wave 1) and adolescents’ later psychological functioning in 11th grade (Wave 4) were partially mediated by parental self-efficacy and parenting practices when the adolescents were in 8th grade (Wave 3). Path analyses were conducted in MPlus v. 7.4. Among 660 Black American families from the Maryland Adolescent Development in Context Study, adolescents’ positive perceptions of school climate were directly related to fewer internalizing and externalizing symptoms, higher resourcefulness, and higher self-esteem. Adolescents’ perceptions of school climate were also indirectly related to their externalizing symptoms through parent–adolescent communication and conflict. Adolescents who reported more positive school climates reported higher parent–adolescent communication and lower parent–adolescent conflict. Adolescents’ reports of school climate were also indirectly associated with self-esteem. Parents’ perceptions of school climate were not directly related to adolescents’ psychological functioning but were directly related to parental self-efficacy and parent–adolescent communication. They were also indirectly related to adolescents’ externalizing symptoms through parental self-efficacy. Parental self-efficacy was positively related to parent–adolescent communication and parents’ home-based school involvement. Overall, the findings highlight the role of school context in adolescents’ psychological functioning and family processes.

## 1. Introduction

School climate refers to the perceived quality of the school environment and is a multidimensional construct that includes the academic environment, interpersonal relationships, safety, and the institutional organization (Franco et al., 2023; Wang & Degol, 2016). School climate has been found to relate to adolescents’ outcomes such as feelings of academic achievement, academic engagement, and psychological functioning (Aldridge & McChesney, 2018; Wang & Degol, 2016; Wong et al., 2021). Despite the significant amount of time children spend in schools, parents’ interactions with schools, and ecological models that highlight how children’s immediate environments can influence one another (Bronfenbrenner & Morris, 2007), little research has focused on how school climate is related to family processes.

Due to the history of anti-Black racism and segregation in the United States, Black American parents often struggle to find schools that meet their children’s needs, including academic rigor, safety, positive interpersonal relationships, fair discipline, and a supportive environment for identity development (Posey-Maddox et al., 2021). Black American students also report more negative perceptions of school climates compared to other ethnicities (Bear et al., 2017; Fisher et al., 2020). Parents’ schooling concerns may also be heightened during their children’s adolescence as their children typically experience multiple school transitions, have increased autonomy, and are more likely to experience exclusionary discipline (Benner, 2011; Eccles et al., 2013). Ongoing challenges in children’s schooling may affect Black American parents’ sense of effectiveness and parenting practices, influencing their children’s psychological functioning. Therefore, in this study we examined how both Black American parents’ and adolescents’ perceptions of school climate were related to parental self-efficacy, parenting, and adolescents’ psychological functioning. Whereas school climate has been directly linked to adolescents’ psychological functioning (Aldridge & McChesney, 2018), we posit that school climate can also influence adolescents’ psychological functioning through its effects on family processes. We examined these links longitudinally by examining adolescents’ school climate in 7th grade, family processes in 8th grade, and adolescents’ psychological outcomes in 11th grade.

### 1.1. Theoretical Framework

This paper is grounded in Bronfenbrenner and Morris’ (2007) bioecological model, which posits that human development occurs based on proximal processes, or reciprocal interactions, over time between an individual and their environments. These proximal processes include parent–child, teacher–student, and peer–peer interactions that occur in children’s immediate environments, or microsystems, including families and schools. The effects of proximal processes on development differ based on the characteristics of the person and environments, including the characteristics of other people such as parents. Most children primarily spend time with their families and at school. The interaction between these two microsystems constitutes a mesosystem, where school and home dynamics influence each other and shape children’s outcomes. Children’s characteristics, such as gender and academic aptitude, may also influence these processes and outcomes. In addition, parents’ characteristics such as education, marital status, and parental self-efficacy (i.e., how effective they think they are at parenting) may shape how parents respond to their perceptions of school climate. This theory suggests that adolescents’ school climate can impact parent–adolescent interactions, including communication and conflict, as well as school–family interactions like parental involvement. Together, school climate, family processes, and characteristics of the child and parent can shape adolescent psychological functioning.

In addition, the integrative model for the study of developmental competencies in minority children (Garcia-Coll et al., 1996) discusses that children’s social position characteristics such as race and gender can lead to them experiencing social stratification processes including racism, prejudice, discrimination, and segregation. These various types of social stratification can shape whether children’s environments, including schools, are promotive, inhibiting, or both for development. For example, Black students are more likely to attend schools that have high rates of poverty (de Brey et al., 2019; Reardon et al., 2024). These schools tend to have fewer resources and teachers with lower skills, which are both inhibiting for learning (Garcia-Coll et al., 1996; Reardon et al., 2024). Differences between parents’ and schools’ values and expectations can inhibit children’s performance and development (Garcia-Coll et al., 1996). Black parents often report more teacher and administrator discouragement of their school involvement and racialized school practices towards their children, which can increase school distrust (Lewis & Diamond, 2025; Posey-Maddox et al., 2021). On the other hand, schools that have appropriate resources for learning, cohesive values and goals with families, and positive socioemotional environments can promote children’s development (Garcia-Coll et al., 1996). One path through which promotive and inhibiting environments shape children’s competencies is through their impacts on families, including families’ goals and beliefs, and parenting practices (Garcia-Coll et al., 1996).

### 1.2. School Climate and Adolescents’ Psychological Functioning

Adolescence is a key period in which to study relations between school climate and adolescent psychological functioning. Studies show that students’ perceptions of school climate lower in middle school and high school (Bear et al., 2017; Wang & Dishion, 2012). In addition, there are significant concerns about adolescent mental health, with 15.1% of teenagers in the United States experiencing major depressive episodes and 36.7% reporting feelings of hopelessness (Bitsko et al., 2022).

Aldridge and McChesney (2018) reviewed 48 studies linking school climate to adolescent mental health. They found that most studies found that positive perceptions of school climate, particularly interpersonal relationships, school connectedness, and school safety, were related to better psychological well-being and fewer mental health problems among adolescents. High academic pressure was related to lower psychological functioning whereas promotion of learning was related to better psychological functioning. Students who feel safe and connected to school report better mental well-being (Patalay et al., 2020), and positive teacher–student relationships are linked to improved mental health (Miller-Lewis et al., 2014).

This study focuses on the role of school climate in adolescents’ psychological functioning, specifically internalizing and externalizing symptoms, self-esteem, and resourcefulness. Positive school climates, especially during the transition to secondary school, have also been linked to lower stress, anxiety, and depressive symptoms (Lester & Cross, 2015). Negative perceptions of school climate can contribute to higher externalizing behavior problems and conduct-disordered behaviors among adolescents (S. P. Thomas & Smith, 2004; Wang & Dishion, 2012). Positive school climates have also been found to promote self-esteem among African American adolescents (Fisher et al., 2020). Less research is on resourcefulness, which is one’s ability to overcome difficulties and includes the concepts of self-control, self-direction, problem-solving, and self-efficacy (X. Li et al., 2018; Rosenbaum, 1989, 1990). Aspects of school climate including school belonging and interpersonal relationships have been related to higher adolescent self-efficacy among middle school and high school students (Zysberg & Schwabsky, 2021).

### 1.3. School Climate, Parental Self-Efficacy, and Parenting

Adolescents’ school climates can influence parental self-efficacy and parenting. Parental self-efficacy entails a parent’s beliefs in their ability to successfully guide their children’s development (Bandura, 1997; Glatz et al., 2024). Parental self-efficacy guides parents’ goals and effort to achieve those goals (Bandura et al., 2011; Hoover-Dempsey et al., 2005). It has been found to predict parents’ school involvement, parenting practices, and psychological well-being, and adolescent outcomes such as academic self-efficacy, academic achievement, and psychological well-being (Jones & Prinz, 2005; Smith et al., 2024). Adolescents’ outcomes can be influenced by parental self-efficacy through its influence on parenting behaviors and through parents’ modeling of healthy behavior.

According to Bandura’s social cognitive theory, self-efficacy is not fixed but can vary across domains and situational conditions (Bandura, 2012). Parental self-efficacy often declines during adolescence as parenting goals and tasks shift as their children undergo numerous developmental (pubertal, cognitive, and social development) and contextual changes (e.g., school transitions) (Glatz & Buchanan, 2015; Glatz et al., 2024). Schools can be sources of support or stress during these changes. Bandura (2012) identified several sources of self-efficacy, including mastery experiences, social modeling (i.e., observing similar others succeed), social persuasion, and physical and emotional states (e.g., levels of anxiety and depression) (Bandura, 2012). Schools can affect parents’ experiences of these sources of self-efficacy (Hoover-Dempsey et al., 2005).

First, schools with positive environments increase parents’ opportunities for mastery experiences. Students who attend schools with more positive environments perform better academically and have better socioemotional well-being, which can increase parents’ feelings of effectiveness (Aldridge & McChesney, 2018). Schools with effective communication and collaboration between parents, teachers, and administrators help align family and school goals, making parenting easier and ensuring concerns are addressed (Epstein & Sanders, 2002; M. G. Sanders, 2009). Second, social modeling takes place in schools. Schools that emphasize positive interpersonal relationships often model healthy communication and interpersonal relationship skills to children and their families (Collaborative for Academic, Social, and Emotional Learning, (CASEL, n.d.)). In addition, schools provide opportunities for parents to connect with other families, fostering social networks that help navigate parenting and school challenges (Kosunen & Rivière, 2018; Posey-Maddox et al., 2016). Third, schools can persuade parents by providing information that encourages parents to engage in certain types of activities and practices that can aid in their children’s success (M. G. Sanders, 2009; CASEL, n.d.). Finally, schools with positive environments can help alleviate parents’ fears about their children’s safety and academic success, whereas schools with negative school climates can increase stress (Soliman et al., 2018).

In addition, research has linked school climate to parenting practices. Models of parental school involvement highlight the important role of school climate in increasing parent involvement (Hoover-Dempsey & Sandler, 1997; Hoover-Dempsey et al., 2005). Parents’ positive perceptions of their children’s middle school climate are linked to beliefs about being actively involved in their children’s education (Whitaker & Hoover-Dempsey, 2013), and greater school involvement (Gale et al., 2024). In contrast, Black parents have also engaged in more school involvement when their children experience discrimination in contexts where they perceive poor parent–teacher relationships (Rowley et al., 2010). These findings suggest that while a positive school climate can boost parental involvement and shape their beliefs, negative school experiences may prompt parents to adopt protective strategies to protect their children. School climates may also influence parent–adolescent interactions by influencing stress levels. Negative school climates can increase stress for adolescents and parents, which can spill over into family interactions (Repetti & Bai, 2025), contributing to higher conflict and lower communication.

### 1.4. Parental Self-Efficacy and Parenting Practices

Numerous studies have examined the role of parental self-efficacy in shaping parenting behaviors (Albanese et al., 2019). In general, high parental self-efficacy has been linked to increased maternal involvement at home (Lemmons, 2015) and open family communication within families (Bandura et al., 2011; Glatz et al., 2018). Studies have found parental self-efficacy to be a strong predictor of parenting styles and strategies. More specifically, higher parental self-efficacy is associated with more responsive and promotive parenting styles (Aranda, 2014; Glatz & Buchanan, 2015) and less negative parenting practices (Bor & Sanders, 2004; Laforce, 2004; M. R. Sanders & Woolley, 2005). Despite these consistent findings, research on the relationship between parental self-efficacy and parenting in African American families remains limited. Ardelt and Eccles (2001) found parental self-efficacy to be a stronger predictor of effective parenting in African American families than in White American families. These findings highlight the need to further examine the direct relationship between parental self-efficacy and parenting, particularly parental academic support and delinquency prevention during adolescence, a critical period for shaping future developmental outcomes.

### 1.5. Parenting and Adolescent Psychological Functioning

Parents play a critical role in shaping how their adolescents develop (Cox & Paley, 1997). The way parents interact with, discipline, and provide support to their adolescents may impact their adolescents’ psychological functioning (Albanese et al., 2019). Previous research has shown that positive and healthy maternal parenting practices are linked with better adolescent developmental outcomes (Morris et al., 2017).

Navigating the transition from childhood to adolescence can shift family dynamics as well as create greater conflict within the family unit (Dotterer et al., 2014). Parent–adolescent conflicts can involve academic expectations, academic performance, peer relationships, household roles, and other interpersonal issues, potentially negatively affecting the adolescent’s mental health. A study with 187 Black American families found that younger adolescents experiencing high conflict with their parents reported higher depressive symptoms and engaged in riskier behaviors (Skinner & McHale, 2016). Research conducted on Black female adolescents has shown that family conflict is associated with higher depressive symptoms (Constantine, 2006). Parent–adolescent conflict disrupts the family system and can cause dysfunction not only on the familial level but on the individual level as well, which can be manifested as negative mental health outcomes in adolescents. Previous research shows that increased mother–adolescent conflict is linked to lower GPA, school bonding, and school self-esteem among Black youth (Dotterer et al., 2014).

As adolescents transition into greater independence, parent involvement in their education provides guidance, structure, and encouragement, fostering both academic achievement and personal growth. Parents who are engaged with their children’s education tend to assist their children in fostering high academic motivation and success (Wilder, 2023). Adolescents with high parental support are more likely to enroll in college, whereas those with low parental expectations may struggle with motivation and self-esteem (Hill & Tyson, 2009). General high-quality parent involvement is also associated with greater self-esteem, well-being, and fewer depressive symptoms and externalizing symptoms (Cosso et al., 2022). Mother involvement is linked to fewer internalizing and externalizing problems, as well as higher prosocial behavior, among Black adolescents (Profe & Wild, 2015).

Parent–child communication also plays a role in adolescents’ psychological functioning. Black parents’ communication quality is negatively related to adolescents’ internalizing symptoms (Ahn et al., 2021) and positively related to their psychosocial functioning, including self-esteem and problem-solving (Xiao et al., 2011). Previous research has also found that parenting communication is also related to adolescents’ problem-solving effectiveness (Offrey & Rinaldi, 2017). Positive parenting practices assist in facilitating a healthy dynamic within families and influences how children develop their own internal models, which can influence how they think, feel, and act throughout their lives (Bowlby, 2010; Sameshima et al., 2020).

### 1.6. Current Study

**Figure 1:**
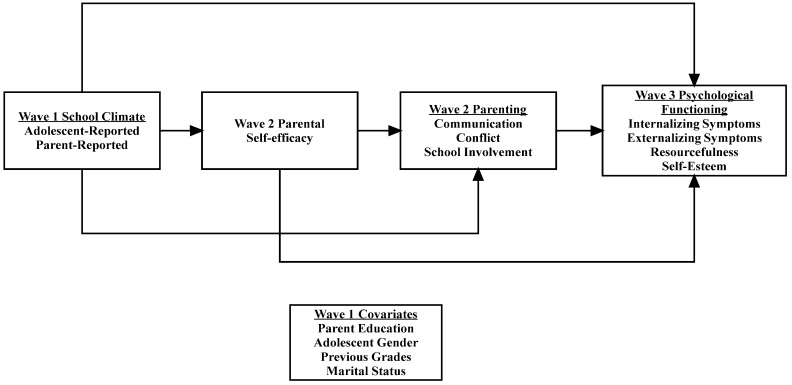
Conceptual model.

In the current study we conducted a secondary analysis to examine whether parents’ and adolescents’ perceptions of school climate when adolescents were in 7th grade were related to adolescents’ 11th grade psychological functioning, including internalizing and externalizing symptoms, resourcefulness, and self-esteem. We also examined whether parental self-efficacy and parenting when their adolescents were in 8th grade mediated the associations between perceptions of school climate during adolescents’ 7th grade and psychological functioning in 11th grade. We predicted that positive perceptions of school climate would be directly related to adolescents’ psychological functioning and that there would be significant indirect relationships through parental self-efficacy and parenting. In addition, we hypothesized that there would be significant indirect effects from school climate to parenting through parental self-efficacy. We tested both adolescents’ and parents’ perceptions of the school climate, as previous work suggests that there is often discordance between youth and parents’ reports. We also controlled for adolescent gender, adolescents’ previous grades, parental educational attainment, and the primary caregivers’ marital status (see Figure 1 for the conceptual model).

## 2. Materials and Methods

### 2.1. Participants

The current study included Black adolescents and their primary caregivers who participated in Waves 1, 3, and 4 of the Maryland Adolescent Development in Context Study (MADICS) when most adolescents were in 7th, 8th, and 11th grade, respectively. There were originally 921 African American families in Wave 1 but there were 660 Black families in our final sample because of attrition across waves. The mean age of the adolescents was 12.89 (SD = 0.33). In the sample, 47.4% of the adolescents were girls and 52.6% were boys. Of the primary caregivers, 93.3% were women and 6.7% were men. In terms of marital status, 57.2% of the primary caregivers were married, 12.8% were never married, 15.6% were divorced, 11.7% were separated, and 2.5% were widowed. In terms of primary caregiver education, 8.3% had less than a high school diploma, 36.1% completed high school only, 33.2% had some college or vocational training, 12.1% completed college, and 10.5% had post-baccalaureate education.

### 2.2. Procedure

In the MADICS, the primary aim was to examine how social contexts such as schools and families shaped adolescents’ psychosocial and academic development and life trajectories (Eccles, 1997). The study sample included seventh graders who attended Prince Georges County, Maryland, middle schools in fall 1991 and their families. The research team collected data through surveys and interviews from the target adolescents, their primary and secondary caregivers, and older siblings starting in the fall of 1991. All participants provided informed consent before participating in the study. The sample included a large proportion of African American families (61%) from socioeconomically diverse backgrounds. The participants were followed up in summer 1992 (Wave 2), 1993 during adolescents’ eighth grade (Wave 3), and from 1996 to 1997 when adolescents were in 11th grade (Wave 4). Waves 5 through 8 occurred when the original adolescents were adults and occurred 1, 3, 11, and 14 years after high school. Information about the study design can be found at https://garp.education.uci.edu/madics.html (accessed on 7 April 2021). For the purposes of this study, Black American participants were included from Waves 1, 3, and 4, when the adolescents were in middle school and high school. Wave 2 was not included as it was a phone interview with questions that differed from the other waves.

### 2.3. Measures

All measures were based on scales constructed by the MADICS research team, as documented in the MADICS codebook (Eccles, 1997). These measures were guided by theory, as well as exploratory and confirmatory factor analyses conducted by the MADICS research team. Most items were adapted from longitudinal studies and established measures. Measure items are included in the Supplemental Information.

#### 2.3.1. Parents’ Perceptions of School Climate (Wave 1)

Parents’ perceptions of school climate were assessed in 7th grade by 10 items in the primary caregiver self-administered survey when adolescents were in 7th grade (alpha = 0.87). The scale included items from school context measures from Maehr and Midgley (1991), Midgley et al. (2000), and Roeser et al. (1998). These items assessed academic rigor (e.g., “all children are expected to do well in their work”), parent–teacher relations (e.g., “teachers understand parents’ perspective”), child–teacher relations (e.g., “the staff cares about students as individuals”), accessibility of the school to parents (e.g., “parents are encouraged to visit if they have special concerns about their child”), and the youth climate (e.g., “kids feel they belong”). These items were measured on a scale from 1 (strongly agree) to 5 (strongly disagree). Items were reverse-coded and averaged so that higher scores meant a more positive school climate.

#### 2.3.2. Adolescents’ Perceptions of School Climate (Wave 1)

Adolescents’ perceptions of school climate (α = 0.75) were also assessed by 10 items completed by the adolescents in their self-administered survey and face-to-face interview in 7th grade. These items were also modified from school context measures from Maehr and Midgley (1991), Midgley et al. (2000), and Roeser et al. (1998) and have been found to be related to behavior problems in middle school (Sameroff et al., 2004). The items started with the prompt “At the school I go to now…”. These items assessed adolescents’ perceptions of the academic rigor (e.g., “the academic program is very good”), student–teacher relations (e.g., the staff cares about students as individuals), peer relations (e.g., “students are not very friendly”), and satisfaction with school (e.g., “in general I like my school a lot”). The items were on a scale from 1 (strongly agree) to 5 (strongly disagree). Some items were reverse-coded so that higher scores meant a more positive school climate. All items were averaged to create this measure.

#### 2.3.3. Parental Self-Efficacy (Wave 3)

Primary caregivers answered seven items in the Wave 3 (8th grade) self-administered survey on their parental self-efficacy in helping their adolescents academically and helping them to avoid delinquency (α = 0.84). This measure was adapted by the MADICS research team and included items developed by Bandura, Cook, and Eccles for the MacArthur Network on Successful Adolescent Development (Eccles, 1997). Primary caregivers were asked “How much can you do to get your 8th grader” (1) to get good grades in math, (2) to get good grades in other school subjects?, (3) to do his or her homework, (4) to get into good activities outside of school such as music, sports, and tutoring programs or community volunteer activities, (5) to stay out of trouble at school, (6) to stay away from the wrong kinds of kids?, and (7) to not use drugs or alcohol. These items were answered on a scale of 1 (nothing) to 4 (a lot). The items were averaged to create the composite variable. Higher scores meant more efficacy.

#### 2.3.4. Parent–Adolescent Conflict (Wave 3)

Adolescents in 8th grade answered 6 items that were modified from the Philadelphia Family Management Study (Furstenberg et al., 1999) regarding conflict with their parents (α = 0.78). Youth were asked the stem “In your family, how often do you argue about…”. The items included (1) “how you spend time outside of school,” (2) “which friends you can spend time with,” (3) “your grades in school,” (4) “at what age you can date,” (5) “what you can wear,” and (6) “spending money?”. The possible responses were on a scale of 1 (almost never) to 5 (almost always). Items were averaged.

#### 2.3.5. Parent–Adolescent Communication (Wave 3)

Adolescents in 8th grade answered three items regarding communication with their parents (α = 0.79) that were modified from the Philadelphia Family Management Study (Furstenberg et al., 1999). They were asked “How often do the following things happen?”. The items included (1) “You talk to your (parent) about how things are going with your friends,” (2) “You talk with your (parent) about your plans for the future,” and (3) “You talk with your (parent) about problems you are having in school.” The possible responses were on a scale from 1 (almost never) to 6 (almost every day). Items were averaged.

#### 2.3.6. Home-Based School Involvement (Wave 3)

Adolescents in 8th grade answered two questions related to their parents’ home-based school involvement. The items included “Your parent(s) helps you with your schoolwork during the school year,” and “Your parent(s) checks your homework after it’s completed.” These items were adapted from the Philadelphia Family Management Study (Furstenberg et al., 1999) and have been used in studies examining parental school involvement among Black American adolescents (Bhargava & Witherspoon, 2015; Tulagan, 2020). The possible responses were on a scale from 1 (almost never) to 6 (almost every day). These items were strongly correlated (r = 0.65). The items were averaged.

#### 2.3.7. Adolescents’ Internalizing Symptoms (Wave 3)

Adolescents answered 15 items rating how often they felt various internalizing symptoms including sadness, loneliness, and withdrawal. These items were adapted from the Children’s Depression Inventory (Kovacs, 1992). Fourteen items were over the past two weeks on scales from 1 to 3. Sample items include “Things bother me,” “I feel like crying,” “I like being with people,” and “I have trouble sleeping.” The meanings of the scale 1 to 3 differed among the items (e.g., 1 (every night/once in a while) to 3 (almost never/all the time)). One item measuring suicidal ideation was on a scale from 1 (almost never) to 5 (almost always). Items were recoded so that higher scores meant higher internalizing symptoms. The items were standardized and averaged (α = 0.86). 

#### 2.3.8. Adolescents’ Externalizing Symptoms (Wave 4)

Adolescents answered 6 items in 11th grade that were modified from the Derogatis’ (1977) Symptom Check List–90-R regarding their externalizing problems (α = 0.73). Three items had the stem “During the last month (including today), how often have you…” and included (1) “felt so angry you wanted to smash or break something”; (2) “felt you could not control your temper”; and (3) “felt so upset you wanted to hit or hurt someone.” These items were rated on a scale from 1 (almost never) to 5 (almost always). Three items had the stem “In the past two weeks…” and assessed getting into fights, obeying authority, and problems completing schoolwork. These three items were on a scale from 1 to 3, with higher scores indicating more externalizing symptoms. All items were standardized and then averaged. Higher scores meant higher externalizing symptoms.

#### 2.3.9. Adolescents’ Self-Esteem (Wave 4)

Self-esteem (α = 0.68) was assessed by adolescents in 11th grade by three items that were modified from Harter’s (1985) concept of self-worth on a scale of 1 (almost never) to 5 (almost always). The items included (1) “How often do you wish you were different than you are,” (2) “How often would you like to change lots of things about yourself if you could,” and (3) “How often are you pretty sure about yourself?” Items were recorded and averaged so that higher scores represented higher self-esteem.

#### 2.3.10. Adolescents’ Resourcefulness (Wave 4)

Resourcefulness (α = 0.74) consisted of 4 items that were modified from the Philadelphia Family Management Study (Furstenberg et al., 1999). These items included (1) How often are you very good at figuring out problems and then planning how to solve them; (2) How often are you very good at carrying out the plans you make for solving problems; (3) How often are you very good at bouncing back quickly from bad experiences; and (4) How often are you very good at learning from your mistakes. Adolescents answered these questions on a scale of 1 (almost never) to 5 (almost always). Items were recoded so that higher scores equaled higher resourcefulness. The items were averaged.

#### 2.3.11. Covariates (Wave 1)

All covariates were assessed in Wave 1. Primary caregivers were asked “what is your child’s sex?” and responded male (0) or female (1). Grades were measured by asking the adolescents “What grades do you usually get?” with a scale from 1 (mostly Fs) to 5 (mostly As). Parent education was measured based on the item “What is the highest grade of school you have completed?” Primary caregivers reported on their marital status as married, widowed, separated, divorced, or never married. We created a dummy variable for marital status with married as 1 and not married as 0.

### 2.4. Data Analysis Plan

Path modeling in MPlus v 7.4 was used to test the associations between perceptions of school climate in adolescents’ 7th grade, parental self-efficacy and parenting in adolescents’ 8th grade, and adolescents’ psychological functioning in 11th grade. All variables were treated as observed variables. The complex samples and cluster functions, along with maximum likelihood estimation with robust standard errors, were used to address nonindependence of observations in the sample (Muthén & Muthén, 2017). Clusters were based on adolescents’ schools in 7th grade as students attended 23 schools in 7th grade, which could affect perceptions of school climate. Full Information Maximum Likelihood was used to address missing data. Cases that had data missing on the Wave 1 variables (school climate perceptions and covariates) or that were missing data on all variables except Wave 1 variables were not included in the analyses based on MPlus defaults. For indirect effects, the 95% confidence intervals were reported.

## 3. Results

### 3.1. Descriptive Statistics

Descriptive statistics and correlational analyses were conducted (Table 1). Parents’ and adolescents’ perceptions of school climate had a small positive correlation (*r* = 0.14, *p <* 0.001). Parents’ perceptions of school climate were positively related to their parental self-efficacy (*r* = 0.18, *p* = 0.002), adolescent resourcefulness (*r* = 0.09, *p* = 0.03), and adolescents’ grades (*r* = 0.10, *p* = 0.005). Adolescents’ perceptions were negatively related to internalizing symptoms (*r* = −0.12, *p* = 0.011), externalizing symptoms (*r* = −0.17, *p* < 0.001), and parent–adolescent conflict (*r* = −0.12, *p* = 0.008) and positively related to resourcefulness (*r* = 0.16, *p* < 0.001), self-esteem (*r* = 0.10, *p* = 0.022), and their grades (*r* = 0.17, *p* < 0.001). Adolescents with married parents also had more positive school climate perceptions (*r* = 0.10, *p* = 0.003). Parental self-efficacy was negatively related to adolescents’ externalizing symptoms (*r* = −0.18, *p* = 0.001) and was positively related to parent–adolescent communication (*r* = 0.13, *p* = 0.003), home-based school involvement (*r* = 0.14, *p* = 0.002), and adolescents’ grades (*r* = 0.14, *p* = 0.003).

Married parents (*r* = 0.10, *p* = 0.038) and parents with higher education (*r* = 0.09, *p* = 0.03) had higher parental self-efficacy. Internalizing symptoms and externalizing symptoms were strongly positively related (*r* = 0.49, *p* < 0.001). Both internalizing and externalizing symptoms were negatively related to resourcefulness and self-esteem. Resourcefulness and self-esteem were positively related (*r* = 0.32, *p* < 0.001). Conflict was positively related to internalizing (*r* = 0.15, *p* = 0.006) and externalizing symptoms (*r* = 0.26, *p* < 0.001) and negatively related to self-esteem (*r* = −0.18, *p* < 0.001). Communication (*r* = −0.17, *p* = 0.001) was negatively related to externalizing symptoms.

### 3.2. Direct Effects

The model fit was χ (2) = 1.479, *p* = 0.477, RMSEA = 0.000, CFI = 1.000, TLI = 1.037, SRMR = 0.008. The model accounted for 5.8% of the variance in parental self-efficacy, 4.3% of the variance in communication, 4.2% of the variance in conflict, 6.1% of the variance in school involvement, 5.6% of the variance in internalizing symptoms, 19.3% of the variance in externalizing symptoms, 7.2% of the variance in resourcefulness, and 6.2% of the variance in self-esteem.

Overall, youth perceptions of school climate were related to each of the measures of psychological functioning (see Table 2 and Table 3). Youth who perceived a more positive school climate reported fewer internalizing symptoms (*b* = −0.10, SE = 0.05, *p* = 0.031), fewer externalizing symptoms (*b* = −0.14, SE = 0.06, *p* = 0.014), higher resourcefulness (*b* = 0.16, SE = 0.05, *p* = 0.001), and higher self-esteem (*b* = 0.14, SE = 0.06, *p* = 0.01). Youth perceptions of school climate were also positively related to parent–adolescent communication (*b* = 0.22, SE = 0.11, *p* = 0.041) and negatively related to parent–adolescent conflict (*b* = −0.15, SE = 0.06, *p* = 0.020) (see Table 4). Adolescents who were in a more positive school climate reported talking more and arguing less with their parents. Youth perceptions of school climate were not significantly related to school involvement (*b* = 0.19, SE = 0.14, *p* = 0.158) or parental self-efficacy (*b* = −0.00, SE = 0.03, *p* = 0.895). In contrast, parents’ reports of school climate were not directly related to adolescents’ psychological functioning or parenting but were positively related to their own parental self-efficacy (*b* = 0.14, SE = 0.05, *p* = 0.008), such that parents who reported more positive perceptions of the school climate also reported higher parental self-efficacy.

Higher parental self-efficacy was related to fewer adolescent externalizing symptoms (*b* = −0.16, SE = 0.06, *p* = 0.003), lower resourcefulness (*b* = −0.20, SE = 0.07, *p* = 0.006), more parent–adolescent communication (*b* = 0.38, SE = 0.14, *p* = 0.007), and more school involvement (*b* = 0.47, SE = 0.15, *p* = 0.001). Higher parent–adolescent conflict was related to higher adolescent internalizing symptoms (*b* = 0.10, SE = 0.04, *p* = 0.022) and externalizing symptoms (*b* = 0.19, SE = 0.06, *p* < 0.001), as well as lower adolescent self-esteem (*b* = −0.17, SE = 0.05, *p* = 0.002). Parent–adolescent communication was related to fewer adolescent externalizing symptoms (*b* = −0.09, SE = 0.02, *p* < 0.001). School involvement was not directly related to adolescents’ psychological functioning.

Girls reported lower resourcefulness (*b* = −0.15, SE = 0.06, *p* = 0.011) and self-esteem (*b* = −0.19, SE = 0.07, *p* = 0.005). Parents of girls also engaged in less school involvement (*b* = −0.44, SE = 0.12, *p* < 0.001). Adolescents with higher grades in 7th grade reported fewer 11th grade externalizing symptoms (*b* = −0.11, SE = 0.04, *p* = 0.008) and had parents with less school involvement (*b* = −0.21, SE = 0.10, *p =* 0.042) and higher parental self-efficacy (*b* = 0.07, SE = 0.03, *p* = 0.013). Parents with higher education had adolescents with higher resourcefulness (*b* = 0.03, SE = 0.01, *p* = 0.001).

### 3.3. Indirect Effects

Table 5 displays indirect effects from school climate perceptions to adolescents’ psychological function and from school climate perceptions to parenting. There was a significant total indirect effect from adolescents’ perceptions of school climate in 7th grade to adolescents’ externalizing symptoms in 11th grade (*b =* −0.04, SE = 0.02, *p =* 0.011, CI 95% [−0.08, −0.01]). There were significant specific indirect effects from youth perceptions of school climate to externalizing symptoms through communication (*b =* −0.02, SE = 0.01, *p =* 0.029, CI 95% [−0.04, −0.002]) and from youth perceptions of school climate to externalizing symptoms through conflict (*b =* −0.03, SE = 0.01, *p =* 0.021, CI 95% [−0.05, −0.004]). Youth who perceived a more positive school climate in 7th grade had higher parent–adolescent communication and lower conflict in 8th grade. Higher parent–adolescent communication and lower parent–adolescent conflict in 8th grade were related to fewer externalizing symptoms in 11th grade. As there was also a significant direct effect from adolescents’ perceptions of school climate to adolescents’ externalizing symptoms, this finding suggests that communication and conflict partially mediated the association between adolescents’ perceptions of school climate and their externalizing symptoms. Family processes overall helped explain the link between school climate and externalizing symptoms.

There were also significant indirect effects between parents’ perceptions of school climate and adolescents’ externalizing symptoms. The total indirect effect was significant (*b =* −0.06, SE = 0.03, *p =* 0.036, CI 95%: [−0.11, −0.003]). One specific pathway that was significant was from parents’ perceptions of school climate to externalizing symptoms through parental self-efficacy (*b =* −0.02, SE = 0.01, *p =* 0.036, CI 95% [−0.05, −0.001]). The direct effect was not significant from parents’ perceptions of school climate to externalizing symptoms. Overall, this pattern of results suggests that pathways through parental self-efficacy explained the association between parents’ perception of school climate and adolescents’ externalizing symptoms.

In addition, there was a significant total indirect effect of youths’ perceptions of school climate on self-esteem (*b* = 0.04, SE = 0.01, *p* = 0.006, CI 95% [0.01, 0.06]). The total effect (*b* = 0.18, SE = 0.06, *p* = 0.002) and the direct effect (*b*= 0.14, SE = 0.06, *p* = 0.010) were significant, which suggests that the model partially explains the link between adolescents’ perceptions of school climate and self-esteem.

There was a significant indirect effect of parents’ perceptions of school climate on parent–adolescent communication through parental self-efficacy (*b* = 0.05, SE = 0.03, *p* = 0.044, CI 95% [0.002, 0.11]). The total (*b* = 0.14, SE = 0.13, *p* = 0.25) and direct effect (*b* = 0.09, SE = 0.12, *p* = 0.437) were not significant. This suggests that parental self-efficacy mediates the link between parents’ perceptions of school climate and parent–adolescent communication. Parents’ perceptions of school climate were also significantly indirectly related to parents’ school involvement through parental self-efficacy (*b* = 0.07, SE = 0.03, *p* = 0.040, CI 95%: [0.003, 0.129]). The total (*b* = 0.16, SE = 0.11, *p* = 0.173) and direct effects (*b* = 0.09, SE = 0.11, *p* = 0.41) of parents’ perceptions of school climate on school involvement were not significant. Together, these findings suggest that parents who perceive a more positive school climate in their children’s school have higher parental self-efficacy, which is related to subsequent higher communication with their children and higher school involvement in the following school year.

## 4. Discussion

In this study we examined links between parents’ and adolescents’ perceptions of the adolescents’ school climate in 7th grade and adolescents’ psychological functioning in high school (11th grade), including internalizing and externalizing symptoms, resourcefulness, and self-esteem. We also examined whether there were indirect pathways between school climate and adolescents’ psychological functioning through parental self-efficacy and parenting. We examined both parents’ perceptions and adolescents’ perceptions of school climate, as there is often discordance in parents’ and adolescents’ reports of parenting and environments (Molinari & Grazia, 2023). Parents’ and adolescents’ perceptions of school climate may differ immensely due to the difference in criteria that they are evaluating (Leadbeater et al., 2015). For example, parents may report based on their perceptions of school safety, teaching quality, relationships with school staff, and their children’s performance. However, children are more likely to report and form perceptions based on their own everyday experiences.

We found that adolescents’ perceptions of their school climate in 7th grade were directly related to their internalizing and externalizing symptoms, resourcefulness, and self-esteem four years later in 11th grade. These were small effects but were consistent with previous findings that linked adolescents’ perceptions of school climate and adolescent psychological functioning (Aldridge & McChesney, 2018). Schools serve as primary social and developmental environments for adolescents, and factors related to school climate, such as sense of belonging, safety, and academic stress, may ultimately impact an adolescent’s mental health (Zysberg & Schwabsky, 2021).

Adolescents’ perceptions of school climate were also indirectly related to their externalizing symptoms through parent–adolescent communication and conflict. To the extent that schools with negative climates are stressful, previous work suggests that stress can hinder family relationships (Repetti & Bai, 2025). In this case, a more negative school environment contributed to more conflict and less communication between parents and adolescents, although these effects were small. Adolescents may communicate less with their parents about their problems if they are embarrassed or concerned about eliciting negative emotional or behavioral reactions or sanctions related to their school experiences (Y. Li et al., 2023). Positive relations between teachers and students can also model positive communication between adolescents and adults, including parents (CASEL, n.d.). Often schools that have a more positive school environment have components that include social emotional learning, which includes healthy communication and conflict resolution skills.

Adolescents’ perceptions of school climate were also indirectly related to their self-esteem. The small indirect effect means that the mediators simultaneously helped explain a small proportion of the variance in this association despite there not being significant specific indirect effects. The lack of significant specific indirect pathways could be due to power, particularly as there was a marginally significant indirect effect from school climate to conflict to self-esteem. Overall, adolescence is a sensitive time for adolescents to develop a strong sense of self as they are more sensitive to both stressors and positive social experiences and undergo significant identity development (Pfeifer & Berkman, 2018; Sisk & Gee, 2022). A positive school climate can help support adolescents’ development of self-esteem through healthy relationships, the development of academic competence, and appropriate levels of autonomy (Eccles et al., 2013; Ryan & Deci, 2017). A positive school climate can support families in engaging in parenting that supports adolescents’ developmental needs. More research is needed to further explore this pathway from adolescents’ perceptions of school climate to self-esteem through family processes.

In contrast, parents’ perceptions were not directly related to adolescents’ psychological functioning. One reason for this may be that parents’ perceptions of school climate were partly based on their perceptions of their children’s experiences. Parents’ and adolescents’ perceptions may also not be strongly aligned if adolescents choose not to disclose their feelings and everyday experiences to their parents (Y. Li et al., 2023). Parents may base their perceptions on secondhand information such as teacher reports, school reputation, and policy (Leadbeater et al., 2015). In addition, parents are likely to have very different interactions than their adolescents with teachers, staff, and administrators. Together, this may explain why adolescents’ perceptions of their school climate were more predictive of their psychological functioning.

Even though parents’ perceptions of school climate were not directly related to adolescents’ psychological functioning, they were indirectly related to adolescents’ externalizing symptoms through parental self-efficacy. In addition, parents’ perceptions of school climate were indirectly related to their home-based school involvement and parent–adolescent communication through parental self-efficacy. These associations were small in meaning. Parents who had more positive perceptions of their adolescents’ school in 7th grade felt more effective than parents in 8th grade. In turn, they had more conversations with their adolescents in 8th grade and were more involved in their schoolwork. Schools that establish positive relationships with parents can develop mutual communication that aligns the school’s and family’s goals for children and communicate strategies to achieve these goals (CASEL, n.d.). This alignment and communication can contribute to parents feeling more efficacy in their parenting (M. G. Sanders, 2009). A positive school climate may also increase parental self-efficacy in cases where parents selected the school their child attends as it may reinforce that they can make decisions that positively influence their children. A positive school climate likely contributes to less stress and fewer barriers that parents must solve. A negative school climate may contribute to a parent feeling more helpless to aid their child in achieving a positive outcome and to address externalizing behaviors.

Parents who feel more efficacious also may feel less threatened in having open communication with their adolescents and engage in more democratic practices, which can increase parent–child communication (Bandura et al., 2011). In addition, when parents are more consistent and supportive, adolescents may be more likely to disclose information about their lives (Y. Li et al., 2023). Parents who feel more effective as parents also likely will feel more capable of helping their child with homework.

There was an interesting, but small, finding that parents with higher parental self-efficacy had youth who reported lower resourcefulness. Parents who have higher parental self-efficacy may engage in more problem-solving to help their adolescents. While this may have the positive intention of helping their children, these adolescents may have fewer opportunities to solve their own problems or make decisions than an adolescent with a less efficacious or involved parent (Givertz & Segrin, 2014; Ungar, 2009). Parents who want to help their adolescents develop more resourcefulness may need to allow them to experience adversity and solve problems. An appropriate level of challenge can help adolescents develop healthy coping skills (Spencer, 2008), particularly with parental support and guidance as needed.

School involvement was not found to be related to adolescents’ psychological functioning. This contrasts with past findings that have found school involvement to be related to adolescent psychological functioning (Barger et al., 2019; Wang & Sheikh-Khalil, 2014). This finding may be related to the measure in this study focusing on parents’ home-based school involvement. It is possible that more comprehensive measures that include parents’ school-based involvement and academic socialization would yield similar results to past studies. Parental home-based school involvement also can have a different meaning to adolescents versus younger children, as parents of adolescents tend to be less involved with homework unless adolescents are having difficulty (Hill & Tyson, 2009; Wang & Sheikh-Khalil, 2014).

## 5. Strengths, Limitations, and Future Directions

This study has several strengths. First, it is a longitudinal study that spans from middle school to high school, a time when perceptions of school climate, parental self-efficacy, and adolescents’ psychological functioning tend to decline (Bear et al., 2017; Bitsko et al., 2022; Glatz et al., 2024). This study also focused on several dimensions of psychological functioning, including mental health problems and psychological well-being, demonstrating that adolescents’ school and family contexts have differing associations with various aspects of adolescents’ functioning. We examined both parents’ and adolescents’ perceptions of school climate, which had different associations with family processes and adolescent psychological functioning. Most importantly, this study provided evidence that schools have significant implications for parental self-efficacy and parenting. Even though social context has been theorized to impact parental self-efficacy and parenting (Bandura, 2012; Bronfenbrenner & Morris, 2007; Garcia-Coll et al., 1996), more studies should explore how both adolescents’ and parents’ contexts influence parental cognitions and behavior. This study also included a socioeconomically diverse sample of Black youth.

There are several limitations to this study. First, the data were collected in the 1990s; however this is one of the few longitudinal studies that has a large sample of socioeconomically diverse Black youth and includes parent and adolescent reports. While there is no evidence that perceptions of school climate may relate differently to parenting and adolescent outcomes today, there has been an increase in school choice mechanisms such as charter schools and voucher programs, which may impact parents’ and adolescents’ perceptions of school climate (Duszka, 2018). Perceptions of school climates may also be lowered by reforms such as an increased emphasis on high-stakes standardized testing (Von der Embse et al., 2016), as well as increased fears of school shootings (Mallett, 2020). In addition, there are more diverse ways that parents can engage with schools, such as through smartphone applications or email (Can, 2016; M. Thomas, 2019), that are not captured in this study’s measures. Contemporary adolescents report more mental health problems, such as anxiety, depression, and suicidal ideation, today than in the 1990s, suggesting that our results may underestimate the level of adolescents’ internalizing symptoms (Mojtabai & Olfson, 2020). In contrast, juvenile delinquency and substance use have declined since the 1990s, which could mean that today’s adolescents have lower externalizing symptoms than those in our sample (Kennedy et al., 2020). These differences in schools and adolescents’ mental health could contribute to different patterns in the associations between school climate, parenting, and adolescent psychological functioning. The significant associations found in this study were small in magnitude, which means that while parents’ and adolescents’ perceptions of school climate are related to adolescents’ subsequent psychological functioning over time, other factors not included in this study may have larger effects and account for more variance in adolescents’ psychological functioning. There are additional parenting variables and aspects of the school environment that could be examined. Parents’ other forms of school involvement and parental restrictiveness are parenting practices that could be influenced by school climate and parental beliefs but were not able to be included in this study because of the availability of measures and the complexity of the model. The reliability of the measure of self-esteem was only moderate, so a more reliable measure may yield stronger effects. There were many statistical tests conducted in this study, which can increase the risk of Type I errors (incorrectly rejecting true null hypotheses). On the other hand, some researchers safeguard against the risk for Type II errors (failure to reject false null hypotheses) through conservative corrections such as the Bonferroni correction (Barnett et al., 2022). Therefore, in this study we discussed all findings that were significant at *p* < 0.05 but caution the reader to interpret these findings with caution (Feise, 2002). Future studies should conduct further investigations into specific adolescent psychological and behavioral outcomes such as externalizing symptoms to further elucidate how family processes are involved in the relations between school experiences and adolescents’ psychological functioning. Finally, although this study is longitudinal, the results are correlational; thus, causation cannot be established.

## 6. Conclusions

Schools are important contexts that impact adolescents’ health and well-being. This study suggests that school climate can have long-term consequences for adolescents’ psychological functioning directly and indirectly through its associations with family processes. School climate can inform parents’ self-efficacy and their interactions with their adolescents. Schools should consider policies and practices that promote positive climates and seek input from parents, helping them feel that the schools are accessible and that teachers and administrators care about their children and their input (CASEL, n.d.). In addition, students need to have positive interactions with teachers, administrators, and peers in schools, as well as experience appropriately high academic rigor and expectations. Middle school is a time when many adolescents start feeling less engaged with school; a positive school climate can help facilitate feelings of school belonging and connection to others that impact their academic and socioemotional trajectories (Eccles et al., 2013). As finding positive school environments for their children is a common stressor for Black families (Posey-Maddox et al., 2021), this study suggests that the role of schools in Black family dynamics should be examined more comprehensively. Additionally, this study found that school climate in middle school was related to adolescents’ psychological functioning in high school. Thus, partnerships between local middle and high schools that focus on improving or aligning school climate, engaging families and local community, facilitating school transitions, and sharing mental health resources may be beneficial for students, their families, and schools.

## Figures and Tables

**Table 1:** Correlations between study variables.

Variable	1.	2.	3.	4.	5.	6.	7.	8.	9.	10.
1. W1 Adolescent School Climate	-									
2. W1 Parent School Climate	0.14 ***	-								
3. W4 Internalizing	−0.12 *	−0.06	-							
4. W4 Externalizing	−0.17 ***	−0.06	0.49 ***	-						
5. W4 Resourcefulness	0.16 ***	0.09 *	−0.21 ***	−0.15 **	-					
6. W4 Self-esteem	0.10 *	0.03	−0.43 ***	−0.23 ***	0.32 ***	-				
7. W3 Parental Self-Efficacy	0.03	0.18 **	−0.04	−0.18 **	−0.08	−0.08	-			
8. W3 Conflict	−0.12 **	−0.08	0.15 **	0.26 ***	−0.08	−0.18 ***	−0.09	-		
9. W3 Communication	0.11 *	0.08	0.01	−0.17 **	0.08	0.02	0.13 **	0.05	-	
10. W3 School Involvement	0.05	0.06	0.07	−0.02	0.02	0.01	0.14 **	0.05	0.47 ***	-
11. W1 Grades	0.17 ***	0.10 **	−0.08 *	−0.23 ***	0.08 *	−0.03	0.14 **	−0.13 **	0.08 *	−0.12 *
12. W1 Parent Education	0.04	0.04	−0.04	−0.08	0.09 *	−0.02	0.09 *	0.02	−0.02	0.02
13. W1 Female ^a^	0.05	−0.01	0.00	−0.14 ***	−0.07	−0.08 *	0.01	0.05	0.11 *	−0.16 ***
14. W1 Married ^a^	0.10 **	0.04	−0.10 *	−0.09 **	0.03	0.01	0.10 *	−0.04	0.09	0.04
N	869	861	526	607	646	648	480	489	489	489
Mean	3.50	3.70	0.02	0.04	3.97	3.87	3.60	2.36	3.64	4.08
SD	0.32	0.25	0.34	0.45	0.44	0.77	0.18	0.67	1.86	2.27

Note: * *p* < 0.05. ** *p* < 0.01. *** *p* < 0.001. W1 = Wave 1. W3 = Wave 3. W4 = Wave 4. ^a^ Dichotomous variable.

**Table 2:** Unstandardized direct effects of internalizing symptoms, externalizing symptoms, and resourcefulness.

	W4 Internalizing Symptoms			W4 Externalizing Symptoms			W4 Resourcefulness		
	*b*	SE	*p*	*b*	SE	*p*	*b*	SE	*p*
W1 Adolescent School Climate	−0.10	0.05	0.031 *	−0.14	0.06	0.014 *	0.16	0.05	0.001 **
W1 Parent School Climate	−0.05	0.05	0.313	0.03	0.05	0.549	0.07	0.06	0.211
W3 Parental Self-Efficacy	0.00	0.08	0.999	−0.16	0.06	0.003 **	−0.20	0.07	0.006 **
W3 Conflict	0.10	0.04	0.022 *	0.19	0.06	<0.001 ***	−0.04	0.03	0.143
W3 Communication	−0.01	0.02	0.517	−0.09	0.02	<0.001 ***	0.06	0.03	0.073
W3 School Involvement	0.03	0.03	0.235	0.01	0.02	0.507	−0.01	0.03	0.631
W1 Female	0.05	0.06	0.427	−0.12	0.06	0.054	−0.15	0.06	0.011 *
W1 Grades	−0.01	0.04	0.721	−0.11	0.04	0.008 **	0.04	0.04	0.308
W1 Parent Education	−0.01	0.01	0.240	−0.01	0.01	0.300	0.03	0.01	0.001 **
W1 Married	−0.06	0.07	0.391	−0.05	0.05	0.314	0.01	0.06	0.907

Note: * *p* < 0.05. ** *p* < 0.01. *** *p* < 0.001. W1 = Wave 1. W3 = Wave 3. W4 = Wave 4.

**Table 3:** Unstandardized direct effects for self-esteem and parental self-efficacy.

	W4 Self-Esteem			W3 Parental Self-Efficacy		
	*b*	SE	*p*	*b*	SE	*p*
W1 Adolescent School Climate	0.14	0.06	0.010 *	−0.00	0.03	0.895
W1 Parent School Climate	0.04	0.07	0.517	0.14	0.05	0.008 **
W3 Parental Self-Efficacy	−0.20	0.13	0.113	-	-	-
W3 Conflict	−0.17	0.05	0.002 **	-	-	-
W3 Communication	0.05	0.03	0.087	-	-	-
W3 School Involvement	−0.00	0.04	0.938	-	-	-
W1 Female	−0.19	0.07	0.005 **	−0.01	0.03	0.686
W1 Grades	0.01	0.05	0.792	0.07	0.03	0.013 *
W1 Parent Education	−0.01	0.02	0.758	0.01	0.01	0.286
W1 Married	0.00	0.07	0.977	0.06	0.04	0.153

Note: * *p* < 0.05. ** *p* < 0.01. W1 = Wave 1. W3 = Wave 3. W4 = Wave 4.

**Table 4:** Unstandardized direct effects for school involvement, communication, and conflict.

	W3 School Involvement			W3 Communication			W3 Conflict		
	*b*	SE	*p*	*b*	SE	*p*	*b*	SE	*p*
W1 Adolescent School Climate	0.19	0.14	0.158	0.22	0.11	0.041 *	−0.15	0.06	0.020 *
W1 Parent School Climate	0.09	0.11	0.408	0.09	0.12	0.437	−0.09	0.10	0.370
W3 Parental Self-Efficacy	0.47	0.15	0.001 **	0.38	0.14	0.007 **	−0.13	0.08	0.106
W1 Female	−0.44	0.12	<0.001 ***	0.26	0.12	0.023 *	0.14	0.06	0.025 *
W1 Grades	−0.21	0.10	0.042 *	0.03	0.07	0.719	−0.11	0.06	0.091
W1 Parent Education	0.01	0.02	0.554	−0.02	0.03	0.510	0.02	0.02	0.283
W1 Married	0.13	0.14	0.328	0.12	0.14	0.391	−0.05	0.08	0.550

Note: * *p* < 0.05. ** *p* < 0.01. *** *p* < 0.001. W1 = Wave 1. W3 = Wave 3.

**Table 5:** Unstandardized indirect effects of climate on adolescents’ psychological functioning and parenting.

				95% Confidence Intervals	
	*b*	SE	*p*	Lower Bound	Upper Bound
W1 ASC → W4 INT (total indirect)	−0.01	0.01	0.289	−0.03	0.01
W1 PSC → W4 INT (total indirect)	−0.01	0.02	0.593	−0.04	0.02
W1 ASC → W4 EXT (total indirect)	−0.04	0.02	0.011 *	−0.08	−0.01
W1 ASC → W3 COM → W4 EXT	−0.02	0.01	0.029 *	−0.04	−0.002
W1 ASC → W3 CONF → W4 EXT	−0.03	0.01	0.021 *	−0.05	−0.004
W1 PSC → W4 EXT (total indirect)	−0.06	0.03	0.036 *	−0.11	−0.003
W1 PSC → W3 PSE → W4 EXT	−0.02	0.01	0.036 *	−0.05	−0.001
W1 PSC → W3 PSE → W3 COM → W4 EXT	−0.01	0.00	0.040 *	−0.01	0.00
W1 ASC → W3 RES (total indirect)	0.02	0.01	0.168	−0.01	0.04
W1 PSC → W3 RES (total indirect)	−0.02	0.02	0.298	−0.05	0.015
W1 ASC → W4 SE (total indirect)	0.04	0.01	0.006 **	0.01	0.06
W1 ASC → W3 CONF → W4 SE	0.03	0.01	0.049 *	0.000	0.049
W1 PSC → W4 SE (total indirect)	−0.00	0.03	0.894	−0.07	0.06
W1 ASC → W3 PSE → W3 COM	−0.00	0.01	0.893	−0.03	0.02
W1 PSC → W3 PSE → W3 COM	0.05	0.03	0.044 *	0.002	0.11
W1 ASC → W3 PSE → W3 INV	−0.00	0.02	0.895	−0.03	0.029
W1 PSC → W3 PSE → W3 INV	0.07	0.03	0.040 *	0.003	0.129
W1 ASC → W3 PSE → W3 CONF	0.00	0.00	0.897	−0.01	0.01
W1 PSC → W3 PSE → W3 CONF	−0.02	0.01	0.175	−0.05	0.01

Note: Only significant specific indirect effects are listed for paths between school climate and psychological functioning. The specific and total indirect effects are the same for paths between school climate and parenting. ASC = adolescents’ perceptions of school climate. COM = communication. CONF = conflict. EXT = externalizing symptoms. INT = internalizing symptoms. INV = school involvement. PSC = parents’ perceptions of school climate. PSE = parental self-efficacy. RES = resourcefulness. SE = self-esteem. W1 = Wave 1. W3 = Wave 3. W4 = Wave 4. * *p* < 0.05. ** *p* < 0.01.

## Data Availability

Restrictions apply to the availability of these data. Data were obtained from the Murray Research Archive and are available at https://dataverse.harvard.edu/dataset.xhtml?persistentId=doi:10.7910/DVN/TVXUIN (accessed on 7 April 2021) with the permission of Murray Research Archive.

## Supplementary Materials

The following supporting information can be downloaded at https://www.mdpi.com/article/10.3390/bs15070933/s1, File S1: Measure Items.

## Abbreviations

The following abbreviation is used in this manuscript:

MADICS	Maryland Adolescent Development in Context
