# Supplementary material for: School Climate and Black Adolescents’ Psychological Functioning: The Roles of Parental Self-Efficacy and Parenting Practices
Source: Behav Sci (Basel). 2025 Jul 10;15(7):933. doi: 10.3390/bs15070933 (PMC12292296; doi:10.3390/bs15070933)
Supplement: Supplementary file 1 [file behavsci-15-00933-s001.zip › behavsci-3632969-supplementary.pdf]

File S1: Measure Items

All measures are from the Maryland Adolescent Development in Context Study. Additional scale information can be found at https://garp.education.uci.edu/madics---data.html

Wave 1 Adolescent Perceptions of School Climate

Scale: 1=strongly agree, 2=agree, 3=neither agree nor disagree, 4=disagree, 5=strongly disagree

At the school I go to now...

1. the academic program is very good.
2. all the kids are expected to do well in their work.
3. the staff cares about students as individuals.
4. students are not very friendly.
5. the principal and teachers generally do not encourage kids like they should.
6. kids generally do good school work.
7. there is good discipline.
8. the kids generally feel like they belong.
9. I would recommend to other kids that they go to my school.
10. In general, I like school a lot.

Wave 1 Parent Perceptions of School Climate

Scale: 1=strongly agree, 2=agree, 3=neither agree nor disagree, 4=disagree, 5=strongly disagree (all items were reverse coded)

Please think about the school your 7th grader is attending this year...

1. all the children are expected to do well in their work.
2. the staff care about students as individuals.
3. children generally feel that they belong.
4. I would be comfortable meeting with my 7th grader's current teacher(s).
5. it is easy to make appointments with teachers and the principal.
6. the teachers understand the parents' points of view.
7. the principal understands the parents' points of view.
8. people with ideas or values different from the commonly accepted ones get a chance to be heard.
9. parents are encouraged to visit if they have special concerns about their child.
10. the teachers and staff work hard to get parents involved.

Wave 3 Parental Self-efficacy

Scale from 1 = nothing, 2 = a little, 3 = some, 4 = a lot

How much can you do to get your 8th grader…

1) to get good grades in math?
2) to get good grades in other school subjects?
3) to do his or her homework?
4) to get into good activities outside of school such as music, sports, and tutoring programs or community volunteer activities?
5) to stay out of trouble at school?
6) to stay away from the wrong kinds of kids?
7) to not use drugs or alcohol?

Wave 3 Conflict

Scale: 1 = almost never, 2 = once in a while, 3 = sometimes, 4 = often, 5 = almost always.

"In your family, how often do you argue about...".

1. how you spend time outside of school.
2. which friends you can spend time with.
3. your grades in school .
4. what age you can date.
5. what you can wear.
6. how you spend money.

Wave 3 Communication

Scale from 1 = almost never, 2 = less than one time a month, 3 = one to three times a month, 4 = one time a week, 5 = a few times a week, 6 = almost every day

1. You talk to your (parent) about how things are going with your friends
2. You talk with your (parent) about your plans for the future
3. You talk with your (parent) about problems you are having in school

Wave 3 Home-Based School Involvement

Scale from 1 = almost never, 2 = less than one time a month, 3 = one to three times a month, 4 = one time a week, 5 = a few times a week, 6 = almost every day

1. Your parent(s) helps you with your schoolwork during the school year
2. Your parent(s) checks your homework after it's completed.

Wave 4 Self-esteem

Scale: 1 = almost never, 2 = once in a while, 3 = sometimes, 4 = often, 5 = almost always.

1. How often do you wish you were different than you are?
2. How often would you like to change lots of things about you if you could?
3. How often are you pretty sure about yourself?

Wave 4 Internalizing Symptoms

Scales from 1 to 3 (response options are in parentheses)

1. I think (I can never be as good as other kids, I can be as good as other kids if I want to, I am just as good as other kids)
2. I feel like ... (I hate myself, I do not like myself, I like myself)
3. I think that… (all bad things are my fault, many bad things are my fault, bad things are usually not my fault)
4. I am worthless… (all the time, many times, once in a while)
5. I feel like crying… (every day, many days, once in a while)
6. Things bother me… (all the time, many times, once in a while)
7. I have trouble sleeping… (every night, many nights, almost never)
8. I feel like… (nobody really loves me, I am not sure if anybody loves me, I am sure that somebody loves me)
9. I am sad… (once in a while, many times, all the time)
10. I feel like… (1 = nothing will work out for me, 2 = I am not sure if things will work out for me, 3 = things will work out for me OK)
11. I do... (most things ok, many things wrong, everything wrong)
12. I think... (I look ok, there are some bad things about my looks, I look ugly)
13. I feel alone… (almost never, many times, all the time)
14. I feel… (I have plenty of friends, I have some friends but I wish I had more, I do not have any friends)

Scale from 1 = almost never, 2 = once in a while, 3 = sometimes, 4 = often, 5 = almost always

15. During the last month, how often have you had thoughts of ending your life?

Wave 4 Externalizing Symptoms

During the last month (including today) how often have you..."

Scale: 1 = almost never, 2 = once in a while, 3 = sometimes, 4 = often, 5 = almost always.

1. felt so angry you wanted to smash or break something?
2. felt you could not control your temper?
3. felt so upset you wanted to hit or hurt someone?

"In the past two weeks..." (response options in parentheses)

4. I find that (I have to push myself all the time to do my schoolwork, I have to push myself many time to do my schoolwork, doing schoolwork is not a problem).
5. I do what I am told (usually, only once in a while, never).
6. I get into fights with people (almost never, many times, all the time).

Wave 4 Resourcefulness

Scale: 1 = Never, 2 = once in a while, 3 = Sometimes, 4 = Often, 5 = Always.

1. How often are you very good at figuring out problems and then planning how to solve them?
2. How often are you very good at carrying out the plans you make for solving problems?
3. How often are you very good at bouncing back quickly from bad experiences?
4. How often are you very good at learning from your mistakes?
